# Supplementary material for: China can be self-sufficient in maize production by 2030 with optimal crop management
Source: Nat Commun. 2023 May 6;14:2637. doi: 10.1038/s41467-023-38355-2 (PMC10164166; doi:10.1038/s41467-023-38355-2)
Supplement: Supplementary file 1 — Supplementary information [file 41467_2023_38355_MOESM1_ESM.pdf]

Supplementary Information for

China can be self-sufficient in maize production by 2030 with optimal crop management

Ning Luo [1, 2], Qingfeng Meng [1 *], Puyu Feng [3], Ziren Qu [1], Yonghong Yu [1], De Li Liu [4,5], Christoph Müller [2] and Pu Wang [1]

[1] College of Agronomy and Biotechnology, China Agricultural University, 100193 Beijing, China.

[2] Potsdam Institute for Climate Impact Research (PIK), Member of the Leibniz Association, 14412 Potsdam, Germany.

[3] College of Land Science and Technology, China Agricultural University, 100193 Beijing, China

[4] NSW Department of Primary Industries, Wagga Wagga Agricultural Institute, Wagga Wagga, NSW 2650, Australia.

[5] Climate Change Research Centre and ARC Centre of Excellence for Climate Extremes, University of New South Wales, Sydney, NSW 2052, Australia.

* Corresponding author: Qingfeng Meng. Email: mengqf@cau.edu.cn

Content the supplementary information

Supplementary figures for main text 2

Supplementary tables for main text 7

References for the supplementary information 11

Supplementary figures for main text

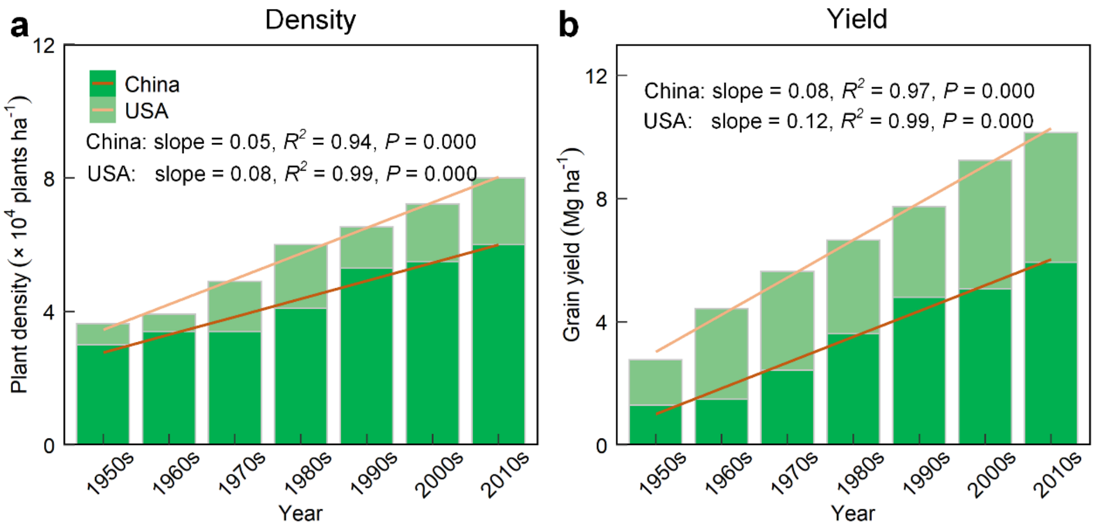

Supplementary Figure 1. Plant density (a) and grain yield (b) in China and USA since 1950s. Data were derived from public dataset[1, 2] (grain yield) and literatures[3, 4] (plant density). Statistical significance is obtained with a two-tailed Student's *t*-test. Source data are provided as a Source Data file.

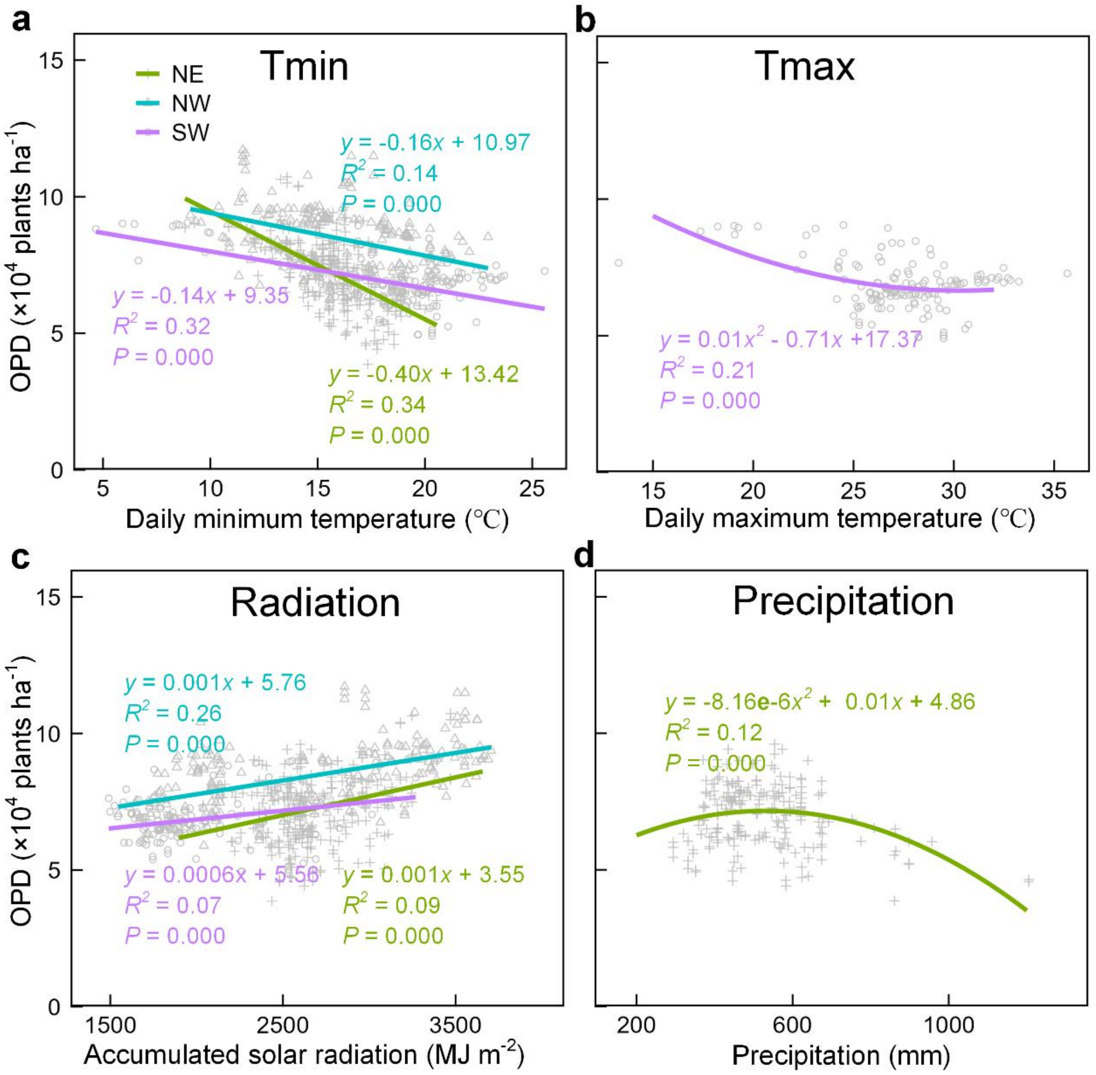

Supplementary Figure 2. Relationships between OPD and climate variables, daily minimum temperature (a), daily maximum temperature (b), accumulate solar radiation (c) and precipitation (d) in maize growing season. Observations based on the literatures (2000-2021) and RF projection in the 2030s. The study area was divided in to four regions: Northeast China (NE), North China Plain (NCP), Northwest China (NW) and Southwest China (SW). Statistical significance is obtained with a two-tailed Student's *t*-test. Source data are provided as a Source Data file.

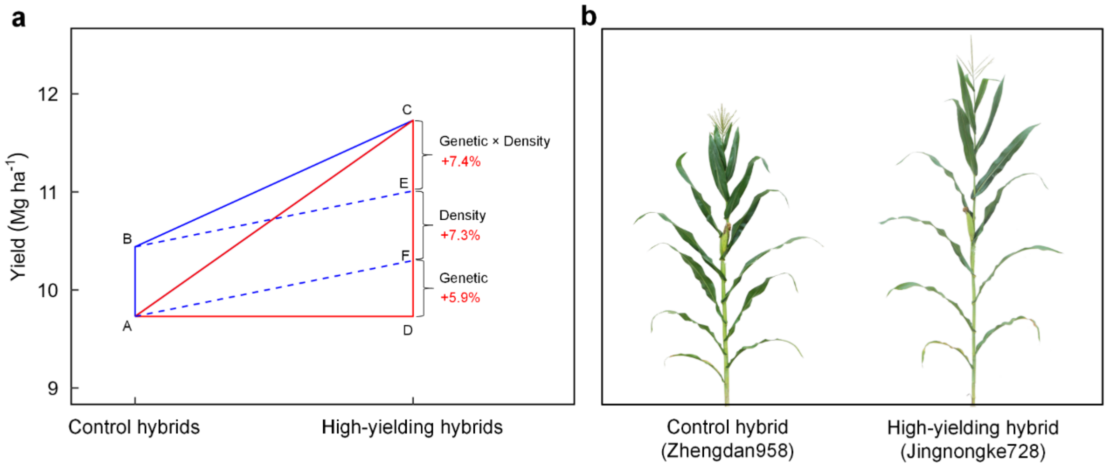

Supplementary Figure 3. Yield improvements from genetics, density and interaction of the both. a, The yield improvement of maize in field observations (87-trials) from the contribution of the genetic (DF), planting density (EF) and density-genetic interaction (CE)[5, 6]. A (or D) represented the control: using the local maize hybrid and farmers' density. B was the local hybrid grown in a high-density. C represented the optimum treatment (OT): OPD with high-yielding maize hybrids. E was the yield of high-yielding maize hybrids with increased density. F indicated the high-yielding hybrids at the farmers' planting density. b, the plant morphology of a control hybrid (Zhengdan958) and high-yielding hybrid (Jingnongke728). Source data are provided as a Source Data file.

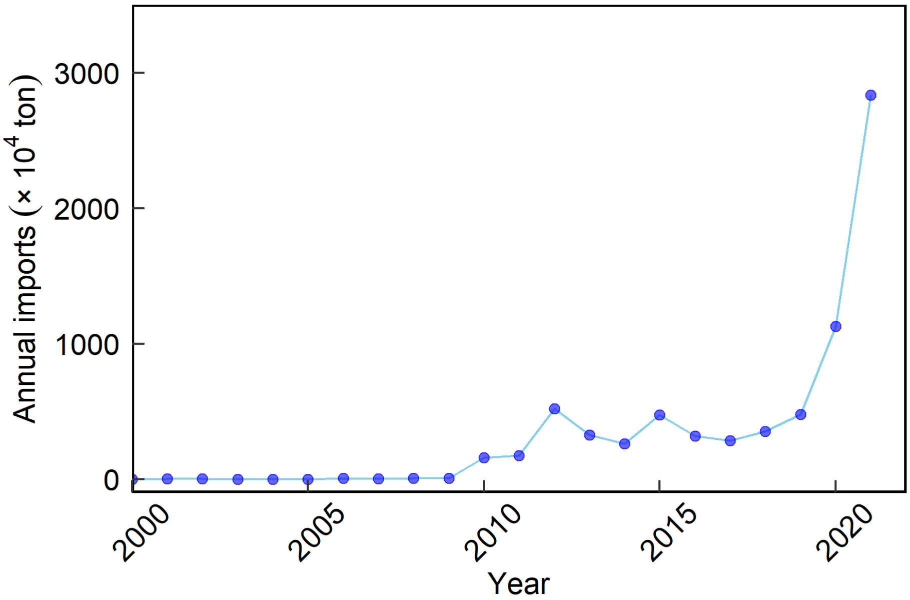

Supplementary Figure 4. China's annual maize imports. (data was extracted from General Administration of Customs. P. R. China; http://english.customs.gov.cn). Source data are provided as a Source Data file.

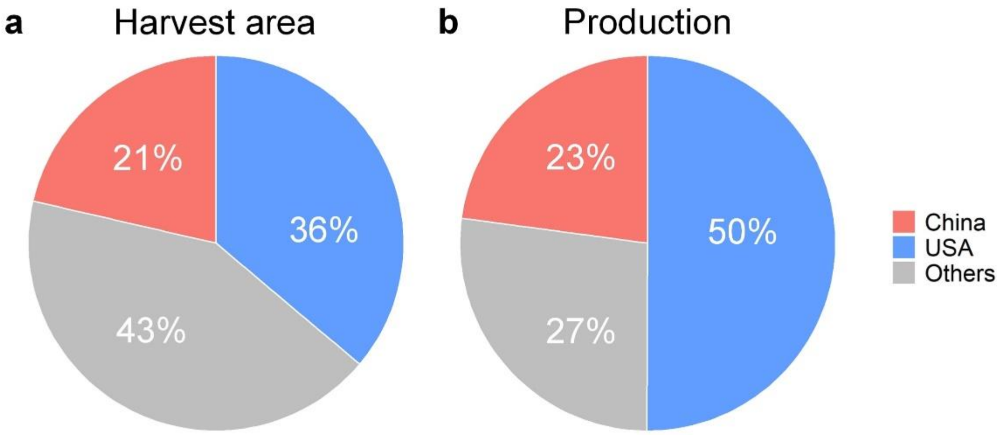

Supplementary Figure 5. Proportion of maize production and harvest area in World for China and the United States. The data are for the latest 5-years (2016-2020) extracted from Food and Agriculture Organization of the United Nations Statistical Database (http://www.fao.org/faostat/en/#data/QC). Source data are provided as a Source Data file.

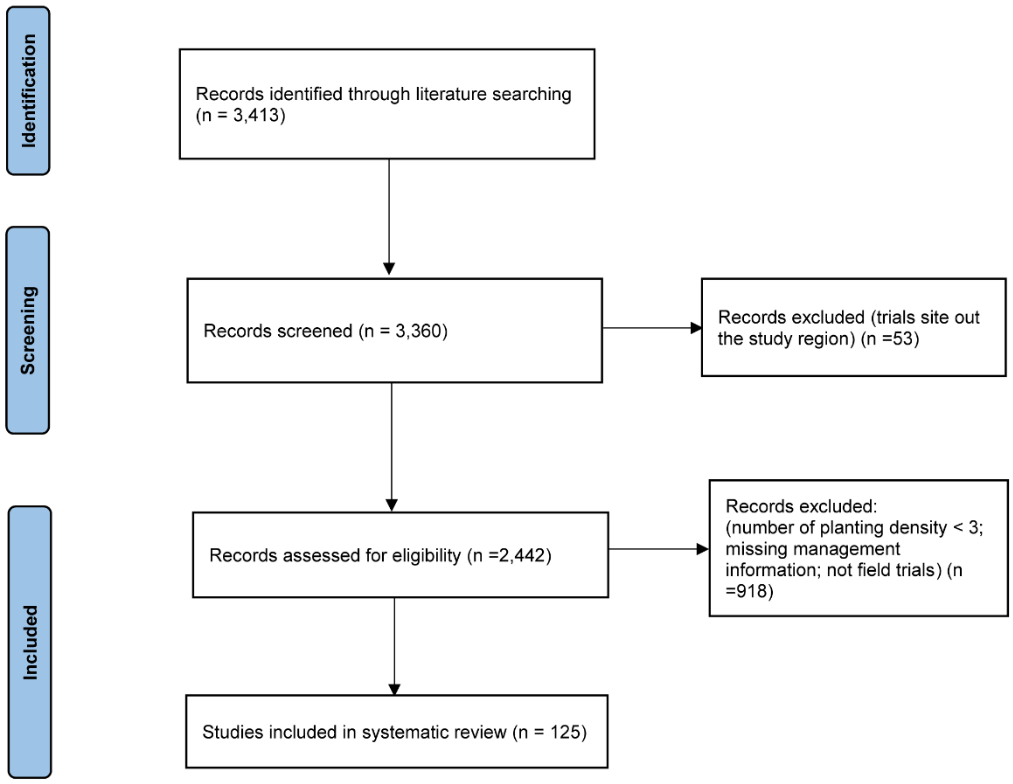

Supplementary Figure 6. PRISMA flowchart showing the process of locating publications included in the present systematic reviews.

Supplementary tables for main text

Supplementary table 1. Descriptive statistics of gaps of planting density and yield between farmers' practice and estimations of RF-model. Note: optimum plant density (OPD) and grain yield $Yield_{OPD}$ projections were simulated based on historical weather in 2010-2019. The study area was divided into four regions: Northeast China (NE), North China Plain (NCP), Northwest China (NW) and Southwest China (SW).

Region	Density (×10^4 plants ha^{-1})			Yield (Mg ha^{-1})		
	Farmer's practice	OPD	Gap	Farmer's practice	$Yield_{OPD}$	Gap
NE	5.6	7.6	2.0	6.3	11.4	5.2
NCP	6.2	7.9	1.7	5.6	11.8	6.2
NW	6.7	8.6	1.9	7.0	12.6	5.6
SW	4.8	7.1	2.3	5.0	10.9	6.0

Supplementary table 2. Descriptive statistics of optimum plant density and yield for field trials. (NE: Northeast China, NCP: North China Plain, and SW: Southwest China)

	Region	n	Control	Optimum
Density				
($\times 10^4$ plants ha^{-1})	NE	37	6.1	7.7
	NCP	34	6.0	8.3
	SW	16	5.3	6.6
	All	87	6.0	7.8
Yield				
(Mg ha^{-1})	NE	37	10.6	12.5
	NCP	34	9.6	11.9
	SW	16	8.3	9.9
	All	87	9.7	11.7

Supplementary table 3. Descriptive statistics of optimum plant density (OPD) and grain yield ($Yield_{OPD}$) projections for major maize regions based on scenario weather data. The study area was divided into four regions: Northeast China (NE), North China Plain (NCP), Northwest China (NW) and Southwest China (SW).

	Region	2010s	2010s+SOM	2030s	2030s+SOM
Density					
($\times 10^4$ plants ha^{-1})	NE	7.5	7.8	7.4	7.6
	NCP	7.8	8.0	7.5	7.8
	NW	8.7	8.9	8.6	8.8
	SW	7.1	7.1	7.1	7.1
Yield					
(Mg ha^{-1})	NE	11.4	11.7	11.2	11.5
	NCP	11.7	11.9	11.4	11.7
	NW	12.7	12.9	12.6	12.8
	SW	10.9	10.9	10.9	10.9

Supplementary table 4. Factors that limit farmers' planting density increases among the four regions. Northeast China (NE), North China Plain (NCP), Northwest China (NW), and Southwest China (SW).

Region	Limitations						References
	Maximum temperature	Minimum temperature	Solar radiation	Irrigation	Soil	Lodging	
NE		√				√	Ming et al., 2017; Li & Wang. 2008; Lobell et al., 2014; Liu et al., 2017; Wu et al., 2019; Meng et al., 2020; Bu et al., 2015; Li et al., 2012; Xue et al., 2016
NCP	√		√		√	√	
NW				√	√	√	
SW	√				√	√	

Supplementary Table 5. List of 22 GCMs under SSP585 future climate scenarios used in this study for statistical downscaling outputs of the 403 sites over the major maize area in China. Details of the 22 GCMs can be found at https://esgf-node.llnl.gov/search/cmip6/.

**Abbreviation**	**GCM name**	**Institution ID**	**Country**
ACC1	ACCESS-CM2	CSIRO-BOM	Australian
ACC2	ACCESS-ESM1-5	CSIRO-BOM	Australian
BCCC	BCC-CSM2-MR	BCC	China
Can1	CanESM5	CCCMA	Canada
Can2	CanOE	CCCMA	Canada
CNR1	CNRM-ESM2-1	CNRM-CERFACS	France
CNR2	CNRM-CM6-1	CNRM-CERFACS	France
CNR3	CNRM-CM6-1-HR	CNRM-CERFACS	France
ECE1	EC-Earth3	EC-Earth-Consortium	Europe
ECE2	EC-Earth3-Veg	EC-Earth-Consortium	Europe
FGOA	FGOALS-g3	CAS	China
GFD2	GFDL-ESM4	NOAA-GFDL	USA
GISS	GISS-E2-1-G	NASA-GISS	USA
INM1	INM-CM4-8	INM	Russia
INM2	INM-CM5-0	INM	Russia
IPSL	IPSL-CM6A-LR	IPSL	France
MIR1	MIROC6	MIROC	Japan
MIR2	MIROC-ES2L	MIROC	Japan
MPI1	MPI-ESM1-2-HR	MPI-M	Germany
MPI2	MPI-ESM1-2-LR	MPI-M	Germany
MTIE	MRI-ESM2-0	MRI	Japan
UKES	UKESM1-0-LL	MOHC	UK

References for the supplementary information

1. Food and Agriculture Organization of the United Nations. *Crops and livestock products* http://www.fao.org/faostat/en/#data/QCL. Accessed 28 March 2022.
2. National Bureau of Statistics (NBS). *China Municipal Statistical Yearbook* https://data.stats.gov.cn/. Accessed 1 May 2022.
3. Mansfield, B. D. & Mumm, R. H. Survey of Plant Density Tolerance in U.S. Maize Germplasm. *Crop Sci.* **54**, 157-173 (2014).
4. Li, S. & Wang, C. *Innovation and Diffusion of Corn Production Technology* (China Scientific Press, Beijing, 2010).
5. Ma, D., Xie, R., Yu, X., Li, S. & Gao, J. Historical trends in maize morphology from the 1950s to the 2010s in China. *J. Integr. Agr.* **21**, 2159-2167 (2022).
6. Tollenaar, M. & Lee, E. Yield potential, yield stability and stress tolerance in maize. *Field Crops Res.* **75**, 161-169 (2002).
7. Bu, L. et al. The effect of adapting cultivars on the water use efficiency of dryland maize (Zea mays L.) in northwestern China. *Agric. Water Manage.* **148,** 1-9 (2015).
8. Li, J., Lammerts van Bueren, E. T., Jiggins, J. & Leeuwis, C. Farmers' adoption of maize (Zea mays L.) hybrids and the persistence of landraces in Southwest China: implications for policy and breeding. *Genet. Resour. Crop Evol.* **59,** 1147-1160 (2012).
9. Liu, B., Chen, X., Meng, Q., Yang, H. & van Wart, J. Estimating maize yield potential and yield gap with agro-climatic zones in China Distinguish irrigated and rainfed conditions. *Agric. For. Meteorol.* **239,** 108-117 (2017).
10. Lobell, D. B. et al. Greater sensitivity to drought accompanies maize yield increase in the US Midwest. *Science* **344,** 516-519 (2014).
11. Meng, Q., Liu, B., Yang, H. & Chen, X. Solar dimming decreased maize yield potential on the North China Plain. *Food Energy Secur.* **9,** e235 (2020).
12. Ming, B. et al. Changes of maize planting density in China. *Sci. Agric. Sin.* **50,** 1960-1972 (2017).
13. Wu, A., Hammer, G. L., Doherty, A., von Caemmerer, S. & Farquhar, G. D. Quantifying impacts of enhancing photosynthesis on crop yield. *Nat. Plants* **5,** 380-388 (2019).
14. Xue, J. et al. Effects of light intensity within the canopy on maize lodging. *Field Crops Res.* **188,** 133-141 (2016).
