# Supplementary material for: China can be self-sufficient in maize production by 2030 with optimal crop management
Source: Nat Commun. 2023 May 6;14:2637. doi: 10.1038/s41467-023-38355-2 (PMC10164166; doi:10.1038/s41467-023-38355-2)
Supplement: Supplementary file 2 — Reporting Summary [file 41467_2023_38355_MOESM2_ESM.pdf]

nature portfolio

Corresponding author(s): Qingfeng Meng

Last updated by author(s): Apr 25, 2023

Reporting Summary

Nature Portfolio wishes to improve the reproducibility of the work that we publish. This form provides structure for consistency and transparency in reporting. For further information on Nature Portfolio policies, see our Editorial Policies and the Editorial Policy Checklist.

Statistics

For all statistical analyses, confirm that the following items are present in the figure legend, table legend, main text, or Methods section.

n/a	Confirmed	
☐	☒	The exact sample size (*n*) for each experimental group/condition, given as a discrete number and unit of measurement
☐	☒	A statement on whether measurements were taken from distinct samples or whether the same sample was measured repeatedly
☐	☒	The statistical test(s) used AND whether they are one- or two-sided<br>*Only common tests should be described solely by name; describe more complex techniques in the Methods section.*
☒	☐	A description of all covariates tested
☐	☒	A description of any assumptions or corrections, such as tests of normality and adjustment for multiple comparisons
☐	☒	A full description of the statistical parameters including central tendency (e.g. means) or other basic estimates (e.g. regression coefficient) AND variation (e.g. standard deviation) or associated estimates of uncertainty (e.g. confidence intervals)
☐	☒	For null hypothesis testing, the test statistic (e.g. *F*, *t*, *r*) with confidence intervals, effect sizes, degrees of freedom and *P* value noted<br>*Give P values as exact values whenever suitable.*
☒	☐	For Bayesian analysis, information on the choice of priors and Markov chain Monte Carlo settings
☒	☐	For hierarchical and complex designs, identification of the appropriate level for tests and full reporting of outcomes
☒	☐	Estimates of effect sizes (e.g. Cohen's *d*, Pearson's *r*), indicating how they were calculated

Our web collection on statistics for biologists contains articles on many of the points above.

Software and code

Policy information about availability of computer code

Data collection	No software was used to collect data in this study. The sources have been described in the manuscript.
Data analysis	R version 4.2.0 was used to training model, aggregate and analyze all results, with packages including 'randomForest (4.7-1.1)', 'tidyverse (1.3.2)' and 'Hmisc (4.7-1)'.

For manuscripts utilizing custom algorithms or software that are central to the research but not yet described in published literature, software must be made available to editors and reviewers. We strongly encourage code deposition in a community repository (e.g. GitHub). See the Nature Portfolio guidelines for submitting code & software for further information.

Data

Policy information about availability of data

All manuscripts must include a data availability statement. This statement should provide the following information, where applicable:

- Accession codes, unique identifiers, or web links for publicly available datasets
- A description of any restrictions on data availability
- For clinical datasets or third party data, please ensure that the statement adheres to our policy

The data supporting the findings of this study are available within the paper and its Supplementary Information and source data files. The literature search was performed using the China National Knowledge Infrastructure (https://www.cnki.net) and the Web of Science (http://www.webofknowledge.com). Climate, soil and maize yield are publicly available from the following sources: historically daily weather data directly collected from the China Meteorological Administration (http://www.nmic.cn); the future scenario climate data are at https://esgf-node.llnl.gov/search/cmip6; soil data are available at http://soil.geodata.cn/data; and maize yield

are at https://data.stats.gov.cn. Data for RF-OPD model training and prediction are available at Zenodo repository: https://doi.org/10.5281/zenodo.7857034 . Source data are provided with this paper.

Human research participants

Policy information about studies involving human research participants and Sex and Gender in Research.

Reporting on sex and gender	This is a study focusing on food security and climate change. Sex and gender were not considered in study design.
Population characteristics	This is a study focusing on food security and climate change. Population characteristics were not considered in study design.
Recruitment	This is a study focusing on food security and climate change. Recruitment were not considered in study design.
Ethics oversight	This is a study focusing on food security and climate change. Ethics oversight were not considered in study design.

Note that full information on the approval of the study protocol must also be provided in the manuscript.

Field-specific reporting

Please select the one below that is the best fit for your research. If you are not sure, read the appropriate sections before making your selection.

☐ Life sciences ☐ Behavioural & social sciences ☒ Ecological, evolutionary & environmental sciences

For a reference copy of the document with all sections, see nature.com/documents/nr-reporting-summary-flat.pdf

Ecological, evolutionary & environmental sciences study design

All studies must disclose on these points even when the disclosure is negative.

Study description	The increasing demand for food and feed with population growth and economic development in China raises questions about the feasibility of self-sufficiency for maize production in the existing area. Here, we address this challenge by combining data-driven projections with a machine learning method on data from 402 stations, with data from 87 field experiments across China. A RF (random forest) algorithm is trained and tested at the nationwide level to predict optimal plant density (OPD). Then, RF predictive model is run with the station-scale dataset to assess OPD across China under current conditions (2010s) and future climate (2030s). These projected OPD are compared with results from field trials across China.
Research sample	The literature database was built by collecting China National Knowledge Infrastructure and the Web of Science for relevant papers published between January 2000 and October 2021. After our review, a total of 125 published studies with 151 site-years and 2442 paired observations were considered eligible. Then, based on the quadratic curves, a dataset contains 448 (site × year × hybrid) paired observations were collected for RF model training and further analyses.
Sampling strategy	We reviewed each literature study based on (i) experiments conducted in field; (ii) at a given field trial, more than three levels of plant density evaluated; (iii) soil properties and management information, in particular, water condition, planting and harvest date reported. After the review, a total of 125 published studies with 151 site-years and 2442 paired observations were considered eligible. Previous researches have shown that the quadratic model performs better in depicting maize yield responses to plant density. Here, we used quadratic curves to gain optimum plant density (OPD) and corresponding yield (YieldOPD) in each specific trial. In total, 448 (site × year × hybrid) paired observations were collected, providing as the basis for the development of the Random Forest (RF) model with OPD.
Data collection	N.Luo download historical climate data, soil information data and county-scale maize yield data. P.Feng and D. Liu collected the future scenario data. N.Luo and Z.Qu collecated literature data.
Timing and spatial scale	Timing scale: The literature dataset covers a period from 2000 to 2021 across major maize area in China; 87-field trials are conducted during 2017-2020; Predictions with the RF model focused on 2010s (2010-2019) and 2030s (2030-2039). Spatial scale: The study focus on major maize area in China, including Northeast China, North China Plain, Northwest China and Southwest China. Our study is conducted at the station-level scale.
Data exclusions	Data exclusions only worked during literature data collecting. To better understanding the optimum density in field trials, we did not include studies with less than three density settings and excluded greenhouse or micro-plot studies in literatures.
Reproducibility	Our results can be reproduced through rerunning the RF model with the extended data. We can provide technical help if readers are interested.
Randomization	Our work based on machine learning approach, which takes advantage of the whole dataset. So randomization is not relevant.
Blinding	Complete blinding in literature data collecting process.

Did the study involve field work? ☒ Yes ☐ No

Field work, collection and transport

Field conditions	Field trials with multi-density settings were conducted over three major maize area in China, covering from extends from southern tropical and sub-tropical systems at low latitudes (Southwest China, SW) to cool-temperate systems at high latitudes (Northeast China, NE). Annual mean precipitation is 594 mm for NE, along with an average mean temperature of 4.9 ℃(from −0.5 to 11.1 ◦C). In the North China Plain (NCP), the annual average temperature ranges from 8 to 15 ◦C, and the annual precipitation is ranging from 300 mm to 1000 mm with an average of 500 mm. The annual temperature averages 15–18 ◦C and precipitation is about 1200 mm in SW.
Location	These trials cover three major maize areas (97.5°– 135.1° E, 21.1°– 53.6° N) including Southwest China, North China Plain and Northeast China.
Access & import/export	Field trials were derived from the National Key Research and Development Program of China (2016YFD0300300, P.W.). All trials were managed by local agricultural experts or /and trained extension officers.
Disturbance	No disturbance were caused by the study.

Reporting for specific materials, systems and methods

We require information from authors about some types of materials, experimental systems and methods used in many studies. Here, indicate whether each material, system or method listed is relevant to your study. If you are not sure if a list item applies to your research, read the appropriate section before selecting a response.

Materials & experimental systems

n/a	Involved in the study	
☒	☐	Antibodies
☒	☐	Eukaryotic cell lines
☒	☐	Palaeontology and archaeology
☒	☐	Animals and other organisms
☒	☐	Clinical data
☒	☐	Dual use research of concern

Methods

n/a	Involved in the study	
☒	☐	ChIP-seq
☒	☐	Flow cytometry
☒	☐	MRI-based neuroimaging
