# Supplementary material for: China can be self-sufficient in maize production by 2030 with optimal crop management
Source: Nat Commun. 2023 May 6;14:2637. doi: 10.1038/s41467-023-38355-2 (PMC10164166; doi:10.1038/s41467-023-38355-2)
Supplement: Supplementary file 4 — Description of Additional Supplementary Files [file 41467_2023_38355_MOESM4_ESM.pdf]

File Name: Supplementary Data

Description: literature database and field trials.
